# The Observation of Humoral Responses after Influenza Vaccination in Patients with Rheumatoid Arthritis Treated with Japanese Oriental (Kampo) Medicine: An Observational Study

**DOI:** 10.1155/2012/320542

**Published:** 2012-04-22

**Authors:** Toshiaki Kogure, Naoyuki Harada, Yuko Oku, Takeshi Tatsumi, Atsushi Niizawa

**Affiliations:** ^1^Department of Japanese Oriental Medicine, Gunma Central and General Hospital, Maebashi Gunma 371-0025, Japan; ^2^Department of Internal Medicine, Gunma Central and General Hospital, Maebashi Gunma 371-0025, Japan; ^3^Department of Japanese Oriental Medicine, Kobe Century Memorial Hospital, Japan

## Abstract

*Objective*. The efficacy of influenza vaccination in patients treated with Japanese Oriental (Kampo) Medicine is unknown. The objectives of this study were to observe the efficacy of influenza vaccination in RA patients treated with Kampo. *Methods*. Trivalent influenza subunit vaccine was administered to 45 RA patients who had received Kampo. They were divided into 2 groups: RA patients treated without MTX (“without MTX group”) and treated with MTX (“with MTX group”). Antibody titers were measured before and 4 weeks after vaccination using hemagglutination inhibition assay. *Results*. Geometric mean titers (GMTs) of anti-influenza antibodies significantly increased for all influenza strains. Response to the influenza vaccination in RA patients treated with Kampo was not lower than that of healthy subjects and the response in the “with MTX group” had a tendency to be higher than that in RA patients treated with MTX in the previous study. There was no significant difference in the GMT after 4 weeks between the “with MTX group” and the “without MTX group.” A decreased efficacy in both seroprotection and seroconversion was not found in the “with MTX group.” *Conclusion*. These observations may open the way for further clinical trials to establish the efficacy for the influenza vaccination in RA patients treated with Kampo.

## 1. Introduction

Rheumatoid arthritis (RA) is a systemic autoimmune disease that is associated with immunologic changes in T cells and B cells. In patients with RA, an impaired ability to react to antigens and an increased peripheral blood CD4/CD8 ratio has been observed in T cells [1, 2]. The presence of soluble interleukin-2 (IL-2) receptors in serum has showed T cell activation [2, 3]. Furthermore, T cell receptor rearrangement excision circles measured from T cells from RA patients were substantially lower than those in healthy controls, because the T cell receptor repertoire has been oligoclonal, which suggests on antigen selection and restriction of the repertoire [4]. There is also a decline in the thymic output of T cells. This premature aging of T cells in RA may have very severe effects on vaccine responses, which are well known to decrease with aging [5]. Additionally, the function of regulatory T cells (CD4+, CD25+) may be abnormal in active RA patients, with a lack of suppression of CD4+ or CD8+ T cells [6].

The multiple immunologic effects of the disease process may in part explain why patients with RA are considered immunocompromised and at increased risk of infection [7]. Therefore, although the exact prevalence, morbidity, and mortality of influenza in patients with RA are unknown, a yearly influenza vaccination is recommended [8]. The influenza vaccination is safe and results in protective levels of antiinfluenza antibodies in most RA patients, even when they are treated with prednisone, disease-modifying antirheumatic drugs (DMARDs), or tumor necrosis factor-blocking agents [9, 10].

In Japan, Japanese traditional herbal (Kampo) Medicine, which is covered by national health insurance, is often prescribed in the primary care field and is also applied as an alternative treatment for serious diseases such as RA. Since ancient times, many kinds of Kampo formulae have been used traditionally and are found to be clinically effective for RA treatment. These formulae usually contain components from several medicinal plants that are thought to exert anti-inflammation and immune-regulator effects and are effective for treating RA [11–13]. We have demonstrated that kampo formula possessed antirheumatic effects in vitro and in vivo [14, 15]. Furthermore, we have observed that the administration of kampo formula partially suppressed T cell activation in collagen induced arthritis (CIA) mice [16]. However, the effectiveness of the influenza vaccination in RA patients treated with Kampo remedy is still not known. The purpose of this study is to investigate the response to the influenza vaccination in RA patients treated with Kampo remedy.

## 2. Patients and Methods

### 2.1. Patient's Profile

 Patients who visited our department in 2010-2011 had to fulfill the American College of Rheumatology 1987 revised criteria for the classification of RA and were selected in a random sampling method. All patients had been treated with Kampo formulae, which were often administered to the patients with RA.

### 2.2. Study Design

An observational study design was utilized in this study. Forty-five patients were entered into this design. Patients received the influenza vaccine intramuscularly from October 2010 until January 2011. Immediately before and 4 weeks after vaccination, blood was drawn for the measurement of C-reactive protein levels (CRP), erythrocyte sedimentation rate (ESR), and anti-influenza antibodies. The Disease Activity Score in 28 joints (DAS28) [17] was recorded before and 4 weeks after vaccination. Information on previous influenza vaccinations was obtained from all participants, and adverse effects occurring in the first 7 days postvaccination were recorded. This study was approved by the Ethics Committee of Gunma Central & General Hospital in Aug 2010.

### 2.3. Vaccine

 We used a trivalent influenza subunit vaccine (2010-2011; Daiichi-Sankyo co.ltd Tokyo Japan) containing purified hemagglutinin and neuramidase of the following strains: A/California/7/2009 (H1N1)-like strain (A/H1N1 strain), A/Victoria/210/2009 (H3N2)-like strain (A/H3N2 strain), and B/Brisbane/60/2008-like strain (B strain).

### 2.4. Hemagglutination Inhibition Assay (HIA)

 The HIA was used for the detection of anti-influenza antibodies. HIAs were performed with guinea pig erythrocytes in accordance with standard procedures [18]. The following parameters for efficacy of the vaccination based on the anti-influenza antibody response were evaluated: geometric mean titer (GMT), fold increase in titer, 4-fold titer rise resulting in a postvaccination level of 40 (seroconversion), and titer rise to 40 ≥ (seroprotection). HIA titers 40 are generally considered to be protective in healthy adults [19].

## 3. Results

### 3.1. Patient Characteristics

Forty-five RA patients were administered Kampo treatment. They were divided into 2 groups as follows: 16 RA patients treated without MTX (without MTX group) and 23 RA patients treated with MTX (with MTX group). Patients treated with tacrolimus (TAC) or biologics were excluded from the patients in the without MTX group, and patients treated with biologics were excluded from the patients in both the with MTX and without MTX group. Their characteristics were shown in Table 1.

### 3.2. The Response to the Influenza Vaccination

 Each GMT after 4 weeks vaccination was 78.8 ± 119.7, 35.7 ± 33.6, and 27.3 ± 27.3 in A/H1N1, A/H3N2, and B strain, respectively (Table 2). Response to the influenza vaccination in RA patients treated with Kampo formulae was not lower than that of healthy subjects in previous studies [20, 21]. There was no significant difference in the GMT after 4 weeks between the “with MTX group” and the “without MTX group.” The GMT in the with MTX group was higher than in the without MTX group (Table 2). The response in the with MTX group had a tendency to be higher than that in RA patients treated with MTX in the previous study [21]. Furthermore, we calculated the fold increase as well as the GMT. The mean fold increase in each group was as follows: 6.5, 2.6, and 2.1, respectively (Table 2). The fold increase in the with MTX group also had a tendency to be higher than in the without MTX group, although this was not significant.

### 3.3. Seroprotection and Seroconversion

**Figure 1:**
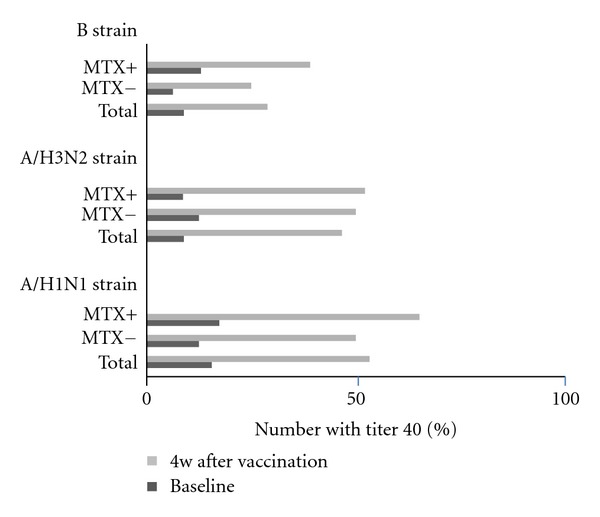
Percentage of patients with anti-influenza titers ≥40, as determined by a hemagglutination inhibition assay for each strain after vaccination with a trivalent influenza subunit vaccine, in total RA patients, RA patients treated with MTX, and RA patients treated without MTX. Solid bars represent prevaccination titer ≥40; open bars represent post vaccination titer ≥40.

 After 4 weeks vaccination, the percentage of patients who possessed the 40 ≥ titer in A/H1N1 was 53.3, 50.0, and 65.2% in total RA patients, without MTX group and with MTX group, respectively (Figure 1). There was no significant difference between the with MTX and the without MTX groups and a decreased efficacy in seroprotection was not found in the with MTX group. In A/H3N2, the percentage of patients who possessed the 40 ≥ titer was 46.7, 50.0, and 52.2%, and in the B strain, 28.9, 25.0, and 39.1% in total RA patients, without MTX group, and with MTX group, respectively. The seroprotection effect observed in the with MTX group had a tendency to be higher than results in the previous study [21]. In seroconversion, the percentage of patients who possessed 40 ≥ titer induced by 4-fold increase was 40.0, 35.6, and 15.6%, respectively (A/H1N1, A/H3N2, and B Strain). There was no significant difference between the with MTX and the without MTX groups also in seroconversion (data not shown).

### 3.4. The Influence of Influenza Vaccination upon RA Disease Activity

The DAS28 did not change after vaccination. There was no adverse reaction by influenza vaccination.

## 4. Discussion

Kampo medicine, which is covered by national health insurance in Japan, is often prescribed in the primary care field, and is also applied as an alternative remedy for RA. The efficacy for RA of Kampo medicines has been demonstrated by case or case series reports and several clinical trials. From these reports, the clinical effectiveness of Kampo therapy is almost similar to that of classical DMARDs, such as bucillamine (Bc) and salazosulfapyridine (SASP). Additionally, several investigators have demonstrated the immunomodulatory effects of Kampo medicine in RA patients as well as an arthritis mouse model, such as CIA [11, 12, 14]. We have also reported that Kampo therapy resulted in a decrease in serum IL-6 levels, but not TNF-*α* levels, as well as the suppression of arthritis development, based on the observations of the CIA mouse model [15]. Furthermore, it has been reported that Kampo medicine is probably effective against infection. The efficacy of Kampo therapy on atypical mycobacterium pneumonia and aspiration bacterial pneumonia has been demonstrated [22, 23], and these effects may be caused by immune-regulator effects, but not direct antibacterial effects. On the other hand, RA patients are susceptible to both viral and bacterial infections. In Japanese RA patients, major causes of death included malignancies (24.2%), respiratory involvement (24.2%) including pneumonia (12.1%) and interstitial lung disease (ILD) (11.1%), cerebrovascular disease (8.0%), and myocardial infarction (7.6%) [24]. Infectious disease is one of the critical factors in the mortality of RA patients. Therefore, a yearly influenza vaccination is recommended by the Center for Disease Control and Prevention (CDC) [25, 26]. However, the immune response to the influenza vaccination has not been reported in RA patients treated with Kampo medicine. This is the first report demonstrating the titer of anti-influenza antibodies before and after influenza vaccination in RA patients administered Kampo formulae.

The response to the influenza vaccination in our population was almost similar to previous results in healthy subjects. Kampo therapy may be beneficial for RA patients from the clinical viewpoint of protection against influenza virus infection as well as suppression of RA disease activity. However, there are various opinions about the efficacy of the influenza vaccination in RA patients. Some reports demonstrate both no differences and significant differences in the response rate between treatment with and without MTX in RA patients [20, 27–29]. This discrepancy may be caused by the different endpoints when measuring the response to the influenza vaccination and different influenza virus roots. Therefore, our data should be limited in reference to the adjuvant effects of Kampo therapy. However, as the baseline titers in this study were less than previous studies, we consider Kampo therapy to be partially beneficial for RA patients in seroprotection and seroconversion. In addition, it has been reported that the response to vaccination was significantly less in patients treated with anti-TNF-a and anti-CD20 antibody (rituximab) drugs than RA patients without biologics [21, 29]. We have checked the titers of the 5 patients treated with biologics, and they were less than those of other RA patient groups (data not shown). Kampo therapy may not influence the response to the influenza vaccination in RA patients treated with biologics. To analyze this problem, further clinical observational studies will be required using a large number of patients.

The RA disease activity by DAS28 did not change after vaccination in our patients. It is generally thought that the vaccination does not influence the disease activity and the titer of the serological markers. A recent report demonstrates that influenza vaccination did not alter the percentage of healthy adults with positive autoantibodies [30].

We have reported several patients with MTX-resistant RA as being successfully treated with Kampo medicine; however, it is still not clear as to how Kampo medicine acts on arthritis in humans [31]. We previously demonstrated that Kampo medicine suppressed polyclonal B cell activation, but not T cell activation, significantly in the CIA mouse model [14, 15]. Recently, it has been clarified that the development of arthritis in the CIA mouse contributed to the differentiation of IL-17 producing cells (Th17), dependent on IL-6 and TGF-*β* [32, 33]. In our previous study using CIA, Kampo medicine decreased the serum IL-6 levels, but not TNF-*α*, suggesting that the suppression of Th17 cell activation by Kampo therapy probably improved the development of arthritis. Thus, we suggest that Kampo medicines do not influence the function of antigen presentation in dendrite cells or macrophages. Based on these findings, we suggest that Kampo therapies do not suppress the response to the influenza vaccination in RA patients. Besides, in innate immunity, we have demonstrated that Juzentaihoto enhanced the production of iNOS in macrophages [34] and the upregulation of NK receptor's expression (Killer-cell immunoglobulin-like receptors) in NK cells [35]. Additionally, the direct anti-influenza virus actions of cinnamon cortex and ephedrae herba (the main herbs composing kampo formulae) have been demonstrated, while these actions are not associated with the response to vaccination in RA patients treated with Kampo [36, 37].

In conclusion, we have demonstrated the changes in the titer of each anti-influenza antibody before and after vaccination in RA patients treated with Kampo formula. A low response to the vaccination was not observed compared with previous studies, and in the MTX-treated patients group, the response to vaccination was higher in our study than in previous reports. The present observations may open the way for further clinical trials to establish the efficacy for the influenza vaccination in RA patients treated with Kampo medicines.

## Figures and Tables

**Table 1:** Characteristics at baseline of RA patients in this study.

	Total	Without MTX group*	With MTX group**
Age, mean ± SD years	56.2 ± 13.5	58.6 ± 10.5	54.1 ± 12.6
No. (%) female/No. (%) male	42 (93)/3 (7)	15 (94)/1 (6)	22 (92)/2 (8)
Duration of RA mean ± SD years	12.2 ± 14.1	13.5 ± 15.6	10.9 ± 11.6
MTX dosage, mean ± mg/week	5.1 ± 3.8	0	7.6 ± 2.5
PSL dosage, mean ± SD mg/day	2.1 ± 2.0	1.6 ± 1.5	2.4 ± 1.9
Taking DMARDs, No.			
Bucillamine	1	1	0
Sulfasalazine	11	8	2
Tacllolimus	4	0	4
DAS28 CRP	3.2 ±1.1	2.9 ±1.0	3.3 ±1.4

*Without MTX group: patients treated with classical DMARDs alone. Patients treated with tacllolimus were excluded. **with MTX group: patients treated with MTX, but not biologics.

**Table 2:** GMTs and fold increase in GMT for influenza A/H3N2, A/H1N1, and B strains in RA patients treated with Kampo formulae before and after administration of influenza vaccines.

	Total	Without MTX group*	With MTX group**
GMT, mean ± SD			
A/H1N1 strain			
Baseline	12.1 ± 14.0	11.0 ± 12.1	14.1 ± 15.0
4 weeks later	78.8 ±119.7	39.6 ± 39.3	115.9 ± 148.8
A/H3N2 strain			
Baseline	13.5 ±13.9	16.0 ±19.7	11.7 ±10.2
4 weeks later	35.7 ±33.6	33.1 ± 21.8	39.1 ± 40.2
B strain			
Baseline	12.8 ±10.3	13.9 ±9.2	11.4 ±11.5
4 weeks later	27.3 ±27.8	22.8 ± 19.2	31.4 ±34.0
Fold increase, mean (range)			
A/H1N1 strain	6.5 (1 to 64)	3.6 (1 to 16)	8.2 (1 to 64)
A/H3N2 strain	2.6 (1 to 16)	2.1 (1 to 8)	3.3 (1 to 16)
B strain	2.1 (1 to 16)	1.6 (1 to 4)	2.7 (1 to 16)

*Without MTX group: patients treated with classical DMARDs alone. Patients treated with tacllolimus were excluded. **with MTX group: patients treated with MTX, but not biologics.
